# Assessment of tilt and decentration of crystalline lens and intraocular lens relative to the corneal topographic axis using anterior segment optical coherence tomography

**DOI:** 10.1371/journal.pone.0184066

**Published:** 2017-09-01

**Authors:** Shuhei Kimura, Yuki Morizane, Yusuke Shiode, Masayuki Hirano, Shinichiro Doi, Shinji Toshima, Atsushi Fujiwara, Fumio Shiraga

**Affiliations:** Department of Ophthalmology, Okayama University Graduate School of Medicine, Dentistry and Pharmaceutical Sciences, Okayama, Japan; Massachusetts Eye & Ear Infirmary, Harvard Medical School, UNITED STATES

## Abstract

**Purpose:**

To investigate the tilt and decentration of the crystalline lens and the intraocular lens (IOL) relative to the corneal topographic axis using anterior segment ocular coherence tomography (AS-OCT).

**Methods:**

A sample set of 100 eyes from 49 subjects (41 eyes with crystalline lenses and 59 eyes with IOLs) were imaged using second generation AS-OCT (CASIA2, TOMEY) in June and July 2016 at Okayama University. Both mydriatic and non-mydriatic images were obtained, and the tilt and decentration of the crystalline lens and the IOL were quantified. The effects of pupil dilation on measurements were also assessed.

**Results:**

The crystalline lens showed an average tilt of 5.15° towards the inferotemporal direction relative to the corneal topographic axis under non-mydriatic conditions and 5.25° under mydriatic conditions. Additionally, an average decentration of 0.11 mm towards the temporal direction was observed under non-mydriatic conditions and 0.08 mm under mydriatic conditions. The average tilt for the IOL was 4.31° towards the inferotemporal direction relative to the corneal topographic axis under non-mydriatic conditions and 4.65° in the same direction under mydriatic conditions. The average decentration was 0.05 mm towards the temporal direction under non-mydriatic conditions and 0.08 mm in the same direction under mydriatic conditions. A strong correlation was found between the average tilt and decentration values of the crystalline lens and the IOL under both non-mydriatic and mydriatic conditions (all Spearman correlation coefficients, r ≥ 0.800; all P < 0.001).

**Conclusion:**

When measured using second generation AS-OCT, both the crystalline lens and the IOL showed an average tilt of 4–6° toward the inferotemporal direction relative to the corneal topographic axis and an average decentration of less than 0.12 mm towards the temporal direction. These results were not influenced by pupil dilation and they showed good repeatability.

## Introduction

An increase in higher-order aberrations caused by tilt and decentration of the intraocular lens (IOL) can lead to deterioration of visual function following procedures such as cataract surgery or suture fixation of the IOL [1]. The accurate measurement of tilt and decentration of the IOL is therefore crucial, and visual function should be assessed based on these measurements.

Thus far, assessments of tilt and decentration of the IOL have been conducted using the Scheimpflug method, the Purkinje method, or anterior segment optical coherence tomography (AS-OCT) [2–10]. All of these methods commonly utilize the pupillary axis under mydriasis as a reference to assess the tilt and decentration of the IOL [2–10]. However, the pupillary axis may not be the optimal reference from which to assess IOL tilt and decentration because it is affected by the shape of the pupil [11,12].

The corneal topographic axis is a reference line that connects the fixation point on the corneal topographer to the corneal vertex [13]. The corneal vertex is not affected by the shape of the pupil, and the corneal topographic axis is therefore considered to be a better reference from which to assess tilt and decentration compared to the pupil center. Indeed, Okamoto et al. reported a significant decrease in higher-order aberrations and coma aberrations as well as a significantly higher contrast sensitivity when using the corneal vertex rather than the pupil center as a reference for applying the laser in wavefront-guided laser refractive surgery [14,15]. However, until recently no testing equipment with the ability to measure the tilt and decentration of crystalline lens and the IOL using the corneal topographic axis as a reference has been available.

In recent years, a second generation of AS-OCT with a 1310-nm laser wavelength has been developed (CASIA2, TOMEY, Japan). This AS-OCT measures the tilt and decentration of the IOL as a reference to the corneal topographic axis. Additionally, this AS-OCT has improved scan rate, scan depth, and scan density, allowing clear imaging of the cornea, the IOL, and the rear surface of the crystalline lens. Therefore, it is now possible to measure the tilt and decentration of not only the IOL but also the crystalline lens with reference to the corneal topographic axis. Thus, in this study, we used second generation AS-OCT to measure the tilt and decentration of both the crystalline lens and the IOL, and we analyzed the measurements for repeatability and the effects of pupil dilation.

## Materials and methods

### Study design and patients

The medical records of 100 eyes of 49 patients were retrospectively reviewed following approval of this study by the Institutional Review Boards of Okayama University Graduate School of Medicine, Dentistry and Pharmaceutical Sciences in June and July 2016. Each patient provided written consent for their medical records to be used in this study, and patient data was accessed anonymously. All eyes had undergone swept-source OCT (CASIA2, TOMEY, Nagoya, Japan) in June or July of 2016. The patient exclusion criteria were as follows: 1) eye problems other than cataracts or high myopia; 2) eye showing phacodonesis or IOL donesis by slit lamp microscopy; 3) history of eye surgeries other than cataract surgery; 4) history of complications during or after cataract surgery; 5) mydriatic pupil diameter of less than 6.0 mm [16,17].

### AS-OCT examinations

All subjects underwent AS-OCT using CASIA2. The CASIA2 uses a 1310 nm swept-source laser wavelength at a frequency of 0.3 seconds, producing 16 AS-OCT images from 16 different angles and a 3-dimensional analysis of the results. Further, it automatically measures the tilt and decentration of the crystalline lens or the IOL relative to the corneal topographic axis. All subjects were measured two times under non-mydriatic conditions and two times under mydriatic conditions. A mixture of 0.5% tropicamide and 0.5% phenylephrine hydrochloride (Mydrin-P, Santen, Osaka, Japan) was used to induce mydriasis. All AS-OCT measurements were taken after at least one week passed following cataract surgery [16]. All measurements were performed by a single ophthalmologist for each subject.

### Cataract surgery

For the 17 patients who underwent cataract surgery, a 2.4 mm self-sealing scleral tunnel incision was made superiorly or at the steepest corneal meridian under local anesthetic. A continuous curvilinear capsulorrhexis measuring approximately 5.5 mm in diameter was created. Following phacoemulsification, the IOL was implanted into the capsule bag with an injector. The capsulorrhexis overlapped the IOL optic in all cases. For all cases, a single-piece IOL was used (W-60, Santen, Osaka, Japan).

### Data analysis

**Fig 1:**
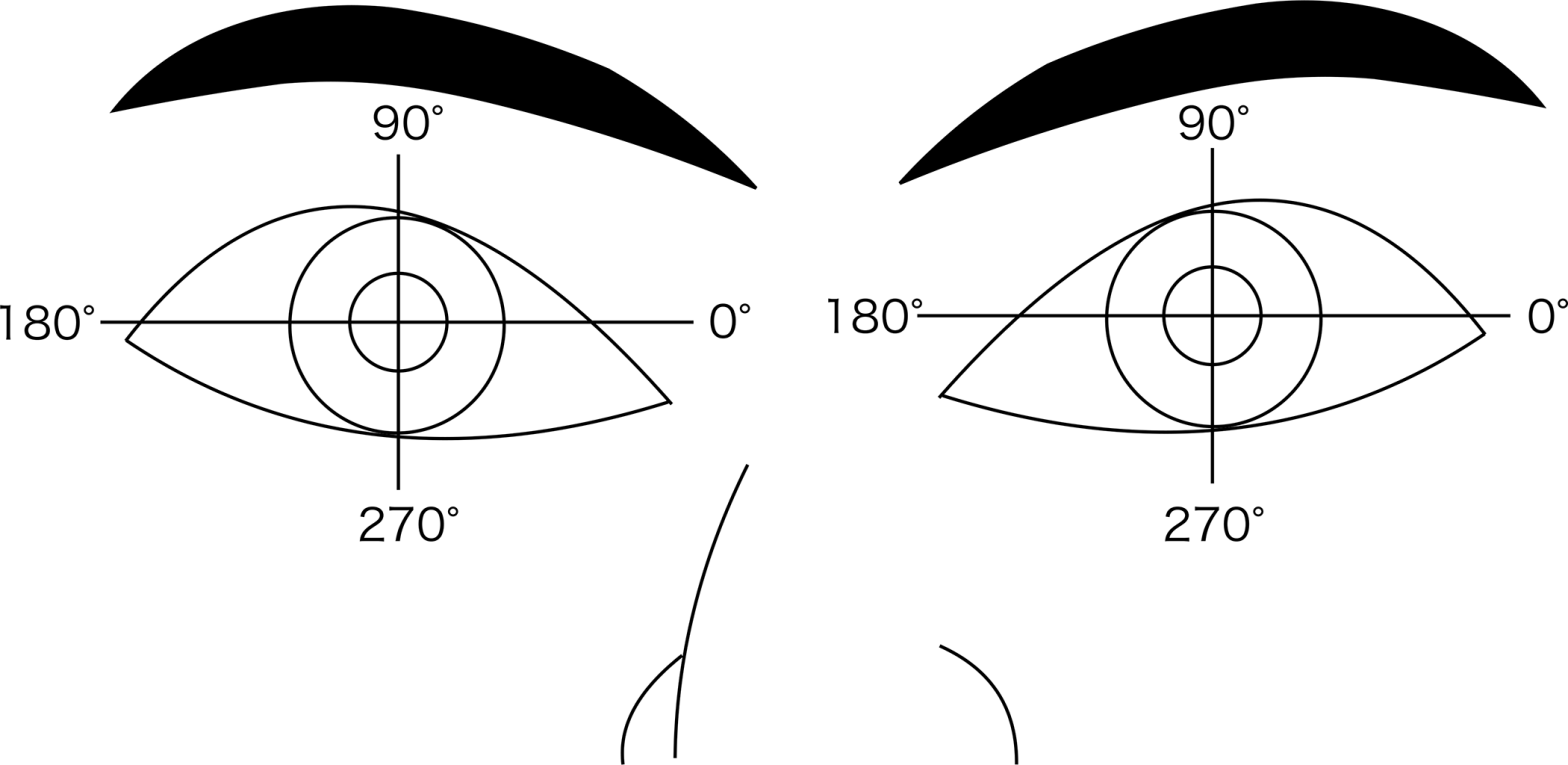
Explanatory diagram of the azimuth for tilt and decentration measurements. The azimuth angle of tilt and decentration for the crystalline lens or the IOL is based on a coordinate system in which the zero degrees is located to the observer's right and 90 degrees is in the superior direction.

All tilt data are presented as “α (degree), β (degree)”, where “α” represents the crystalline lens or IOL tilt from the corneal topographic axis, and “β” represents its azimuth. Decentration data are presented as “γ (mm), Δ (degree)”, where “γ” represents the crystalline lens or IOL decentration from the corneal topographic axis, and Δ represents its azimuth. The azimuth angle for assessing crystalline lens or IOL tilt and decentration utilizes a coordinate system in which the zero-degree direction is to the observer's right and 90 degrees is the superior direction (Fig 1). Individual tilt (degree) or decentration (mm) and its azimuth (degree) values were transformed into Cartesian coordinates (*x* and *y*) using the method described by Holladay et al. [18,19]. Standard descriptive statistics were then applied after this conversion. We also used the method described by Holladay et al. to convert from Cartesian coordinates back to the standard polar notation for tilt or decentration as well as for azimuth. Spearman correlation analysis was used to calculate correlations using non-mydriatic and mydriatic data. Intraclass correlation coefficients (ICC) were calculated to assess repeatability of AS-OCT data. A P value < 0.05 was considered significant. All statistical analyses were performed using SPSS for Windows, version 22.0 (IBM Corp., Armonk, NY, USA). Data are presented as mean ± standard deviation (SD).

### Outcome measures

The main outcome measures were the tilt and decentration of the crystalline lens and the IOL relative to the corneal topographic axis and its azimuth, under both non-mydriatic and mydriatic conditions.

## Results

One-hundred eyes of 49 patients were included in this study. This sample included 25 men and 24 women with a mean age of 73.6 ± 9.0 years (range, 44–90 years). There were 50 right eyes and 50 left eyes included in this study. Imaging data were obtained from the crystalline lens for 41 eyes and from the IOL for the remaining 59 eyes. The mean axial length was 23.40 ± 1.10 mm (Table 1).

**Table 1:** General characteristics of the patient group.

Patient / Eyes (n)	49 / 100
Age, range (years)	73.6 ± 9.0, 44–90
Male / Female (n)	25 / 24
Right eye / Left eye (n)	50 / 50
Right eye, Phakic / IOL	22 / 28
Left eye, Phakic / IOL	19 / 31
Axial length (mm)	23.40 ± 1.1

IOL = intraocular lens

**Fig 2:**
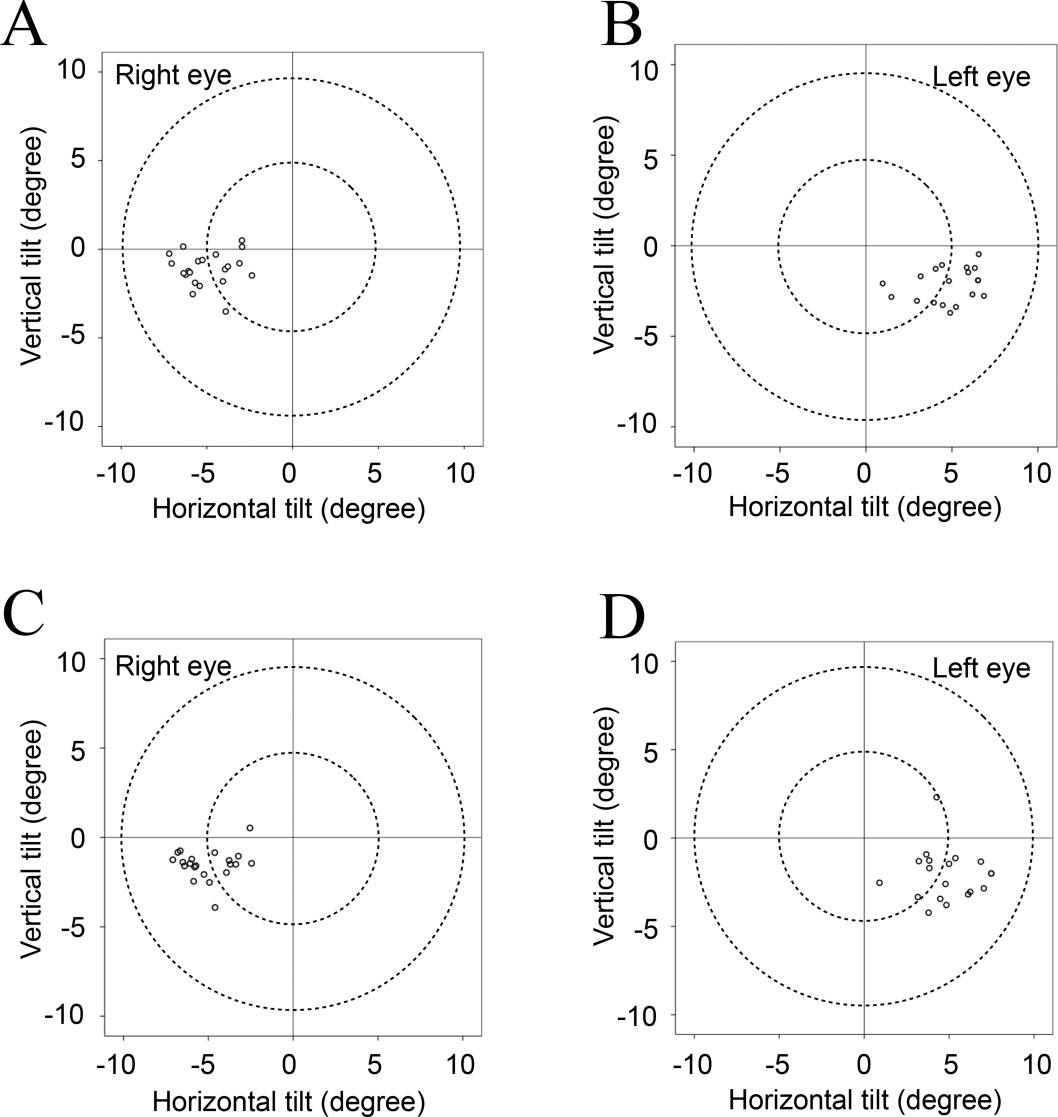
**Tilt of each crystalline lens relative to the corneal topographic axis under non-mydriatic (A, B) and mydriatic (C, D) conditions.** The crystalline lens tended to tilt towards the inferotemporal direction relative to the corneal topographic axis under both non-mydriatic and mydriatic conditions.

**Fig 3:**
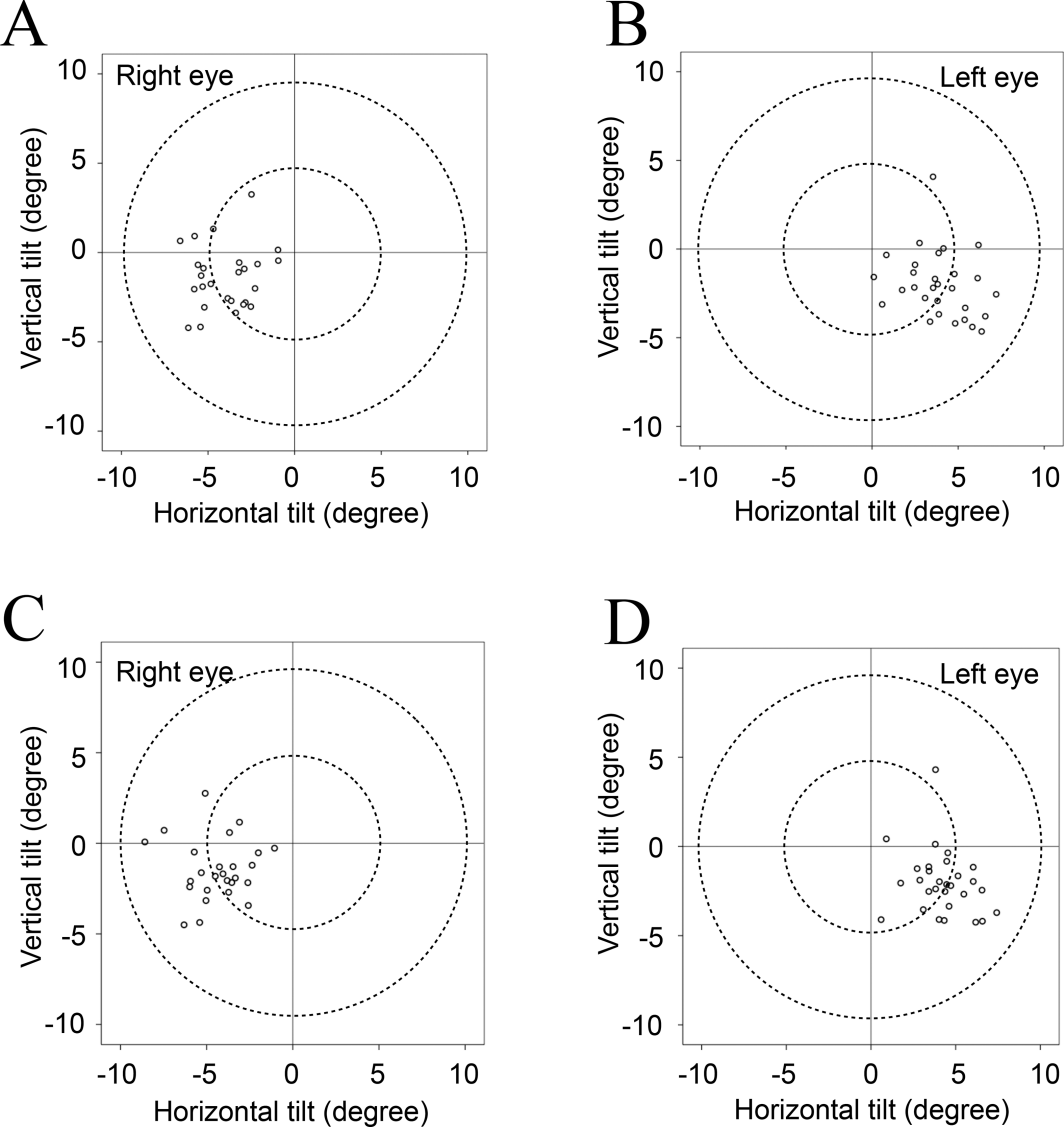
**Tilt of each IOL relative to the corneal topographic axis under non-mydriatic (A, B) and mydriatic (C, D) conditions.** The IOL tended to tilt towards the inferotemporal direction relative to the corneal topographic axis under both non-mydriatic and mydriatic conditions.

**Fig 4:**
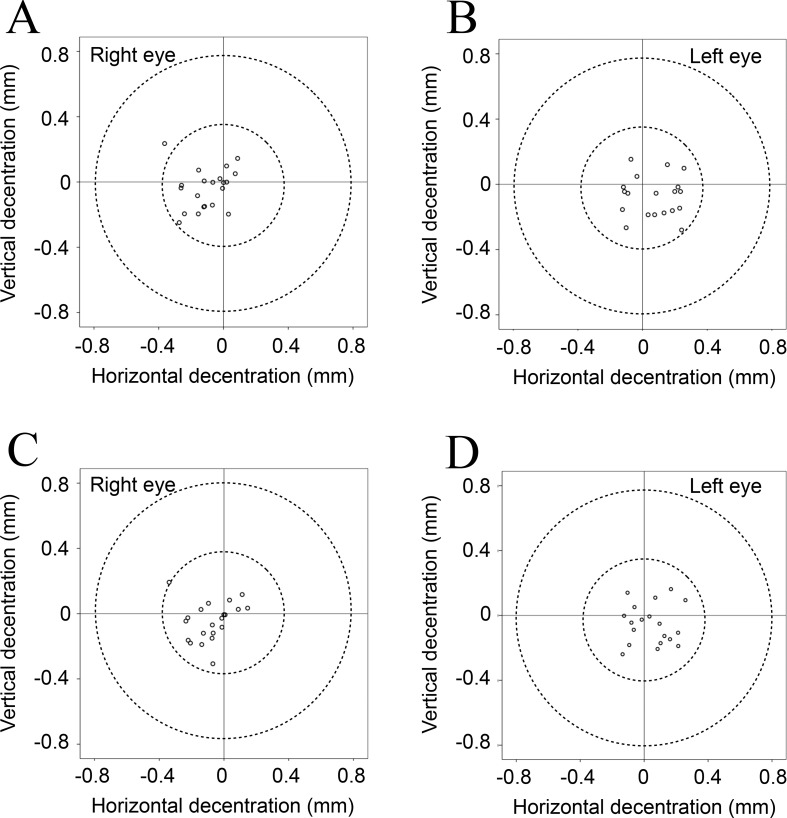
**Decentration of each crystalline lens relative to the corneal topographic axis under non-mydriatic (A, B) and mydriatic conditions (C, D).** The crystalline lens tended to shift towards the temporal direction relative to the corneal topographic axis under both non-mydriatic and mydriatic conditions.

**Fig 5:**
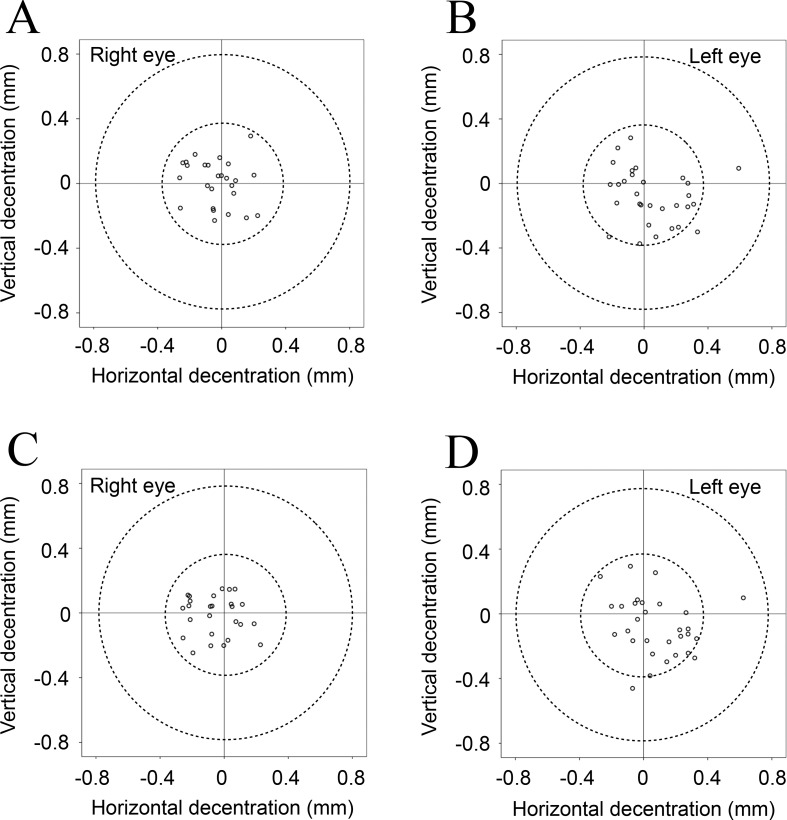
**Decentration of each IOL relative to the corneal topographic axis under non-mydriatic (A, B), and mydriatic (C, D) conditions.** The IOL tended to shift towards the temporal direction relative to the corneal topographic axis under both non-mydriatic and mydriatic conditions.

Table 2 shows the mean values for tilt and decentration of the crystalline lens and the IOL relative to the corneal topographic axis. For the crystalline lens, the mean angle relative to the corneal topographic axis was 5.10° for the right eye and 5.27° for the left eye under non-mydriatic conditions and 5.24° for the right eye and 5.30° for the left eye under mydriatic conditions. For the IOL, the mean angle relative to the corneal topographic axis was 4.22° for the right eye and 4.40° for the left eye under non-mydriatic conditions and 4.62° for the right eye and 4.70° for the left eye under mydriatic conditions. For both the crystalline lens and the IOL, the mean tilt relative to the corneal topographic axis was toward the inferotemporal direction (right eye, 192−200°; left eye, 333−337°). The mean decentration of the crystalline lens and the IOL was 0.03−0.12 mm from the corneal topographic axis under non-mydriatic conditions and 0.06−0.10 mm under mydriatic conditions. The mean decentration for the crystalline lens and the IOL under both non-mydriatic and mydriatic conditions was toward the temporal direction (right eye, 169−209°; left eye, 309−316°). The distribution data for tilt and decentration of the crystalline lens and the IOL under non-mydriatic and mydriatic conditions are shown in Figs 2–5. All relevant data are available in S1 Table.

**Table 2:** Mean tilt and decentration of the crystalline lens and the IOL.

	Tilt, Azimuth		Decentration, Azimuth	
	Non-mydriatic	Mydriatic	Non-mydriatic	Mydriatic
R) Crystalline lens	5.10°, 192°	5.24°, 198°	0.12 mm, 202°	0.09 mm, 209°
R) IOL	4.22°, 200°	4.62°, 199°	0.03 mm, 169°	0.06 mm, 197°
L) Crystalline lens	5.27°, 336°	5.30°, 337°	0.10 mm, 315°	0.07 mm, 309°
L) IOL	4.40°, 333°	4.70°, 334°	0.09 mm, 312°	0.10 mm, 316°

IOL = intraocular lens; L = left; R = right.

We assessed the correlation between mydriatic and non-mydriatic data using both tilt and decentration measurements of the crystalline lens and the IOL. All Spearman correlation coefficients for both lenses under both conditions were found to be greater than or equal to 0.8 (all P values < 0.001; Table 3).

**Table 3:** Spearman correlation between non-mydriatic and mydriatic data.

	Crystalline lens		Intraocular lens	
	*x* axis	*y* axis	*x* axis	*y* axis
Tilt data	0.980*	0.800*	0.961*	0.905*
Decentration data	0.905*	0.908*	0.839*	0.860*

*P < 0.001

Tables 4 and 5 show results from the 17 eyes that were imaged using second generation AS-OCT before and after cataract surgery. Using intraclass correlation coefficients (ICC), significant correlations were found between pre- and postoperative data for both tilt and decentration of the crystalline lens and the IOL under both non-mydriatic and mydriatic conditions (ICC of tilt, 0.733–0.912; all P values ≤ 0.001: ICC of decentration, 0.414–0.559; all P values < 0.05).

**Table 4:** Intraclass correlation coefficients calculated between pre- and postoperative tilt data from the crystalline lens and the intraocular lens.

	*x* axis	*y* axis
Non-mydriatic data	0.912 (P < 0.001)	0.806 (P = 0.001)
Mydriatic data	0.912 (P < 0.001)	0.733 (P < 0.001)

**Table 5:** Intraclass correlation coefficients calculated between pre- and postoperative decentration data from the crystalline lens and the intraocular lens.

	*x* axis	*y* axis
Non-mydriatic data	0.525*	0.485*
Mydriatic data	0.559*	0.414*

*P < 0.05

## Discussion

This study is the first to use AS-OCT to assess the tilt and decentration of the crystalline lens and the IOL relative to the corneal topographic axis. Our results show that both the crystalline lens and the IOL were tilted 4–6° towards the inferotemporal direction relative to the corneal topographic axis and were shifted slightly (less than 0.12 mm) towards the temporal direction.

There have been several studies on the tilt and decentration of the crystalline lens and the IOL thus far [2]. Most of these studies have used the pupillary axis as a reference. According to Eppig et al. [2], the average reported tilt angle for the IOL in previously published papers ranges from 0.87 to 3.43°, the average decentration ranges from 0.23 to 0.34 mm, and no uniform directions for tilt and decentration have been reported. We report a larger mean tilt angle compared to previous studies, an inferotemporal direction of tilt, and a minimal amount of decentration. There are two possible reasons for these discrepancies. Firstly, previous studies have used either the Scheimpflug method or the Purkinje method, and to our knowledge there are no previous studies that have used AS-OCT for detailed analysis of tilt and decentration of the crystalline lens and the IOL. Secondly, this study used the corneal topographic axis as a reference to assess tilt and decentration of the crystalline lens and the IOL, whereas the majority of previous studies have used the pupillary axis. However, a previous report by Baumeister et al. used the optic axis as a reference and reported an average tilt of 3.03−3.26° and an average decentration of 0.23−0.24 mm [17]. Measurements for this study were taken using an EAS-1000, which uses the Scheimpflug method, and data describing direction of tilt or decentration were not presented.

Opinions vary regarding which axis should be used as a reference for evaluation of the IOL as well as for corneal refractive surgery. In previous studies, the corneal vertex has been shown to be a safer and more effective reference for the center of the excimer laser beam in refractive surgery for myopia compared to the center of the pupil [14,15]. Surgeries using the corneal vertex had lower rates of higher-order aberration, coma aberration, and contrast sensitivity deterioration. Additionally, Holladay et al. [20] showed that visual function is affected when the tilt of aspheric lenses is more than 7° or when decentration is more than 0.4 mm. In the present study, measurements of the IOL under mydriatic conditions showed that 7 out of 59 eyes (12%) had a tilt of more than 7°, and 3 eyes (5%) had a decentration of more than 0.4 mm. In our investigation of pre- and post-operative cataract surgery, there was a significant correlation between tilt and decentration for both the crystalline lens and the IOL (Tables 4 and 5). These results suggest that an aspherical lens should not be chosen for the IOL if there is a significant tilt or decentration of the crystalline lens before surgery.

Previous studies on tilt or decentration of the crystalline lens or the IOL have assessed the eyes under mydriasis of more than 5−6 mm [3,5,10,16,21]. In analytical studies using Purkinje images, Guyton et al. [22] and Schaeffel et al. [7] reported tilt and decentration measurements of the IOL obtained without mydriasis; however, the degree of repeatability of these results and whether these measurements were as accurate as those obtained under mydriatic conditions is unclear. Ding et al. [10] reported that measurements using AS-OCT for tilt and decentration have high repeatability compared to analyses using Purkinje images or the Scheimpflug method. We found that tilt and decentration measurements of the crystalline lens and the IOL obtained using AS-OCT had high repeatability under both non-mydriatic and mydriatic conditions (S2 and S3 Tables). These results are clinically significant because they show that it is possible to assess the tilt and decentration of the crystalline lens or the IOL in cases of poor mydriasis using second generation AS-OCT. Second generation AS-OCT is not dependent on pupil diameter, nor is it affected by pupil shape. The present study included only eyes in which no deformation of the pupil was identified. Future studies should investigate the use of second generation AS-OCT for the measurement of tilt and decentration of the crystalline lens and the IOL in eyes with pupillary deformation.

This study has several other important limitations. Briefly, this was a retrospective study; the number of test subjects was small; the type of IOL and the timing of the examination after cataract surgery were not the same for all subjects; and interobserver repeatability was not considered. A large prospective study that accounts for IOL and type and interobserver repeatability is therefore necessary to confirm our results.

In conclusion, this study reveals for the first time that tilt and decentration of the crystalline lens and the IOL can be measured with good repeatability using second generation AS-OCT, regardless of whether the eye is under mydriasis or not.

## Supporting information

S1 Table All relevant data in this manuscript. (XLS)

S2 Table Repeatability of crystalline lens and intraocular lens tilt measurements. (DOCX)

S3 Table Repeatability of crystalline lens and intraocular lens decentration measurements. (DOCX)
