# Supplementary material for: Assessment of tilt and decentration of crystalline lens and intraocular lens relative to the corneal topographic axis using anterior segment optical coherence tomography
Source: PLoS One. 2017 Sep 1;12(9):e0184066. doi: 10.1371/journal.pone.0184066 (PMC5581187; doi:10.1371/journal.pone.0184066)
Supplement: S2 Table — (DOCX) [file pone.0184066.s002.docx]

**Supporting information**

**S2 Table. Repeatability of crystalline lens and intraocular lens tilt measurements**

|  | Crystalline lens | | Intraocular lens | |
| --- | --- | --- | --- | --- |
|  | *x* axis | *y* axis | *x* axis | *y* axis |
| ICC of non-mydriatic data | 0.992* | 0.815* | 0.936* | 0.910* |
| ICC of mydriatic data | 0.995* | 0.906* | 0.995* | 0.956* |

*P < 0.001

ICC = intraclass correlation coefficient
